# The Application of Consensus Weighted Gene Co-expression Network Analysis to Comparative Transcriptome Meta-Datasets of Multiple Sclerosis in Gray and White Matter

**DOI:** 10.3389/fneur.2022.807349

**Published:** 2022-02-24

**Authors:** Keping Chai, Xiaolin Zhang, Huitao Tang, Huaqian Gu, Weiping Ye, Gangqiang Wang, Shufang Chen, Feng Wan, Jiawei Liang, Daojiang Shen

**Affiliations:** ^1^Department of Pediatrics, Zhejiang Hospital, Hangzhou, China; ^2^Department of Neurological Surgery, Tongji Hospital, Tongji Medical College, Huazhong University Science and Technology, Wuhan, China; ^3^College of Life Science and Technology, Huazhong University of Science and Technology, Wuhan, China

**Keywords:** WGCNA, random forest, multiple sclerosis, ssGSEA, RNA-seq

## Abstract

Multiple sclerosis (MS) is a chronic inflammatory disease of the central nervous system characterized by demyelination, which leads to the formation of white matter lesions (WMLs) and gray matter lesions (GMLs). Recently, a large amount of transcriptomics or proteomics research works explored MS, but few studies focused on the differences and similarities between GMLs and WMLs in transcriptomics. Furthermore, there are astonishing pathological differences between WMLs and GMLs, for example, there are differences in the type and abundance of infiltrating immune cells between WMLs and GMLs. Here, we used consensus weighted gene co-expression network analysis (WGCNA), single-sample gene set enrichment analysis (ssGSEA), and machine learning methods to identify the transcriptomic differences and similarities of the MS between GMLs and WMLs, and to find the co-expression modules with significant differences or similarities between them. Through weighted co-expression network analysis and ssGSEA analysis, CD56 bright natural killer cell was identified as the key immune infiltration factor in MS, whether in GM or WM. We also found that the co-expression networks between the two groups are quite similar (density = 0.79), and 28 differentially expressed genes (DEGs) are distributed in the midnightblue module, which is most related to CD56 bright natural killer cell in GM. Simultaneously, we also found that there are huge disparities between the modules, such as divergences between darkred module and lightyellow module, and these divergences may be relevant to the functions of the genes in the modules.

## Introduction

Multiple sclerosis (MS) is a chronic inflammatory disease of the central nervous system characterized by demyelination, which leads to the formation of white matter lesions (WMLs) and gray matter lesions (GMLs) (1, 2). Axon demyelination in different brain regions would lead to distinct symptoms, for example, cerebellar lesions may lead to ataxia, etc. (3, 4). Recently, many studies have shown that although the pathological changes in GMLs and WMLs are both focal demyelinations, the mechanisms of the two are still different. Some hypotheses were put forward to insinuate that varying degrees of immune cell infiltration, such as CD8^+^ *T-*cells, CD4^+^ *T-*cells, and B cells, may play a key role in the progression of the disease. However, the region-specific differences of immune infiltration in MS remain unclear (3, 5–7).

Weighted gene co-expression network analysis (WGCNA) is a classic gene clustering biological method, which relates genes with phenotypes or pathways. It is mainly based on two theories: (1) genes with similar expression patterns may be co-regulated, functionally related, or in the same pathway, and (2) the distribution of gene networks conforms to scale-free. In WGCNA, the weighted value of the correlation coefficient is used, that is, the gene correlation coefficient is taken to the power of N so that the connections among the genes in the network conform to the scale-free network distribution, and this algorithm is more biologically meaningful. It has been widely applied in Alzheimer's disease, Parkinson's disease, cancers, and green halophytic microalgae *Dunaliella salina* (8–13). The consensus network based on WGCNA could discover the similarity and difference of two gene co-expression networks better. It has been performed to measure the similarity of gene expression patterns in the livers of patients with different gender, or in neurodegenerative diseases, such as Alzheimer's disease, Parkinson's disease, etc. (14, 15).

Here, we applied consensus WGCNA and single-sample gene set enrichment analysis (ssGSEA) on the MS transcriptomics data of WM and GM, detected the similarities and differences in the gene expression patterns between WMLs and GMLs, and performed ssGSEA and machine learning analysis to identify the most relevant immune infiltration pathways of MS. Through the analysis above, we obtained the most relevant immune infiltration pathways in GMLs and WMLs (CD56 bright natural killer cell). Furthermore, the most different module between WMLs and GMLs was detected (the darkred and lightyellow modules). We also overlapped the differentially expressed genes (DEGs) in WMLs and GMLs and found that most DEGs in GM were kept in WM DEGs. Overlapped DEGs were mainly distributed in midnightblue, pink, yellow, magenta, and purple modules, which were most similar modules between WMLs and GMLs. Finally, we found that the genes in midnightblue module, such as heat shock proteins (HSPs), may be involved in the pathway in which CD56 bright natural killer cell could resist *T*- and B-cell-associated inflammatory brain damage.

## Methods and Materials

### Data Acquisition and Preprocessing

The data used in this article were obtained from the GEO database GSE123496, which contains five patients with MS (average age = 57.6 years) and five age-matched healthy controls (CON; average age = 56.2 years), and fresh frozen autopsy samples were obtained from Human Brain and Spinal Fluid Research Center in Los Angeles, USA (https://www.ncbi.nlm.nih.gov/geo/query/acc.cgi?acc=GSE123496). Gene expression in the frontal cortex (MS = 5, CON = 5) and parietal cortex (MS = 5, CON = 5) was selected as the gene expression profile in GM, compared with gene expression profile in WM represented by data from an internal capsule (MS = 5, CON = 5) and the corpus callosum (MS = 5, CON = 5). The normalized gene expression matrix data were downloaded, and data filtering was performed before WGCNA. In order to filter data, first, eliminate abnormal samples through hierarchical clustering. Second, eliminate duplicate probes and gene expression data in the expression matrix. Ultimately, there were about 33,000 genes in the dataset used for the follow-up analysis.

### Single-Sample Gene set Enrichment Analysis

The detailed mathematical principle of ssGSEA can be found in Reference (16). The main steps are as follows:

a) First, assuming that there is a sample's expression data, then it should be like this, the first column is the gene, the second column is the expression value, such as a two-column data matrix. The expression levels of all genes in the sample are sorted to obtain their rank among all genes, and the set of these genes is BG.
b) Assuming that we want to analyze the Kyoto Encyclopedia of Genes and Genomes (KEGG), first, we need to find the gmt file corresponding to KEGG on the GSEA official website. The main format of the gmt file is: each line represents a pathway, the first column is the pathway ID, the second column is the description corresponding to the pathway, and the third column to the last column is the gene in the pathway.
c) Then, for any pathway A, we can get the gene list GL of this pathway, find the genes present in BG from GL, count them as NC, and add the expression levels of these genes to SG. ES calculation should be started: for any gene G in the expression profile, if G is a gene in the set GL, then the ES value is equal to the expression level of the gene divided by SG, otherwise, the ES value of the gene is equal to 1 divided by the value of gene set BG total number minus NC. The ES value of the genes in each BG, in turn, should be calculated, and the ES with the largest absolute value as the A.ES of pathway A should be found.
d) After the calculation of the ES value of this pathway A is completed, a statistical method is required to evaluate whether the ES is significant, that is, non-random. According to the above method of calculating ES, first, the expression order of genes in the expression profile should be randomly shuffled, and then the ES value is calculated. It has to be repeated a thousand times to obtain a thousand ES values. According to the distribution of these thousand ES values, to calculate the position of A.ES in this distribution and the probability of appearing in this position, the *p*-value is obtained. The ES and *p*-values of each channel are, in turn, calculated, and then multiple test corrections are used to obtain the false discovery rate (FDR) of each channel.

In this study, the GSVA package based on R 3.4.2 was used as a tool for the ssGSEA analysis, which is a method proposed for ssGSEA (17). The gene sets were acquired from Molecular Signatures Database (MSigDB) according to the description in the section “Introduction,”, and 28 signaling pathways involved immune infiltration were included, such as CD56 bright natural killer cell, CD56 dim natural killer cell, effector memory CD8^+^ T cell, etc.

### Application of Random Forest to Find the key Immune Infiltration Pathway in Classifying the MS and CON in Different Brain Regions

The samples in each brain region were grouped into MS and CON. Inputting the ssGSEA enrichment scores of these 28 immune infiltration pathways in these samples into random forest (RF) classifier *via* sklearn package based on python to predict which group the samples belong to and to identify the most important pathway for classifying.

### Construction of Weighted Gene Co-expression Network and Identification of Significant Modules

The consensus weighted gene co-expression network was constructed by WGCNA package based on R 3.4.2 to identify consensus modules for cross-dataset (GM and WM) comparisons. First, the Pearson correlation coefficient was calculated to assess the similarity of the gene expression profiles, and then the correlation matrices were converted into adjacency matrices. Second, the adjacency matrices between genes were weighted by a soft power (14 for both datasets) function to obtain a scale-free network. The dynamic tree-cut method was used to identify different modules, the adjacency matrix was converted to a topology overlay matrix (TOM), and modules were detected by cluster analysis during module selection. DeepSplit and the minModuleSize adopted the default values. Lastly, the hierarchical cluster was used to identify gene modules, and different modules were represented by different colors.

### Correlation Analysis of Gene Modules With Clinical Phenotype

To detect the associations between modules and clinical phenotypes (ssGSEA scores), first, the match function in WGCNA is used to associate ssGSEA scores with expression matrices. Second, the correlations of the consensus module eigengene (ME) and the ssGSEA scores were calculated by Pearson's analysis. Modules showing significant differences between GM and WM were obtained. Lastly, to further confirm the consensus modules with significant differences in GM and WM datasets, the correlation coefficients between module membership (gene expression level) and gene significance (GS, for assessing the association of genes with phenotypes) were calculated using the labeledHeatmap function, and the *p-*values were obtained.

### Find the Overlapping Genes Between the GM and WM DEGs (Gray Matter MS vs. CON; White Matter MS vs. CON)

The samples were divided into two groups (MS and CON), respectively, in WM and GM, and the Limma packages were preformed to normalize and identify the DEGs (18, 19). Next, using the Venn tool (http://bioinformatics.psb.ugent.be/webtools/Venn/) to discover the overlapping genes between WML and GML DEGs.

### Cross-Validation Criteria for Random Forest

The criteria used for cross-validation are as follows: the data size of the validation set accounts for 25% of the entire data, and the training data account for 75%, which means that a five-fold cross-validation is used.

### The Construction of Protein–Protein Interaction Network

These overlapped genes were also analyzed using GeneMANIA (https://genemania.org/) (20, 21) to construct the co-expression network.

## Results

### Data Preprocessing and Standardization

The GSE123496 dataset includes samples from 5 brain regions, and each sample has 60,234 gene counts (22). We extracted the sample data from different brain regions and divided them into 2 regions roughly (GM and WM, 20 samples in each group, Supplementary Figure 1A). We inputted the gene counts into R for log2 conversion and removed blank and duplicate values, and finally, 32,353 genes were reserved.

### Identify the Overlapped DEGs Between WMLs and GMLs

**Figure 1:**
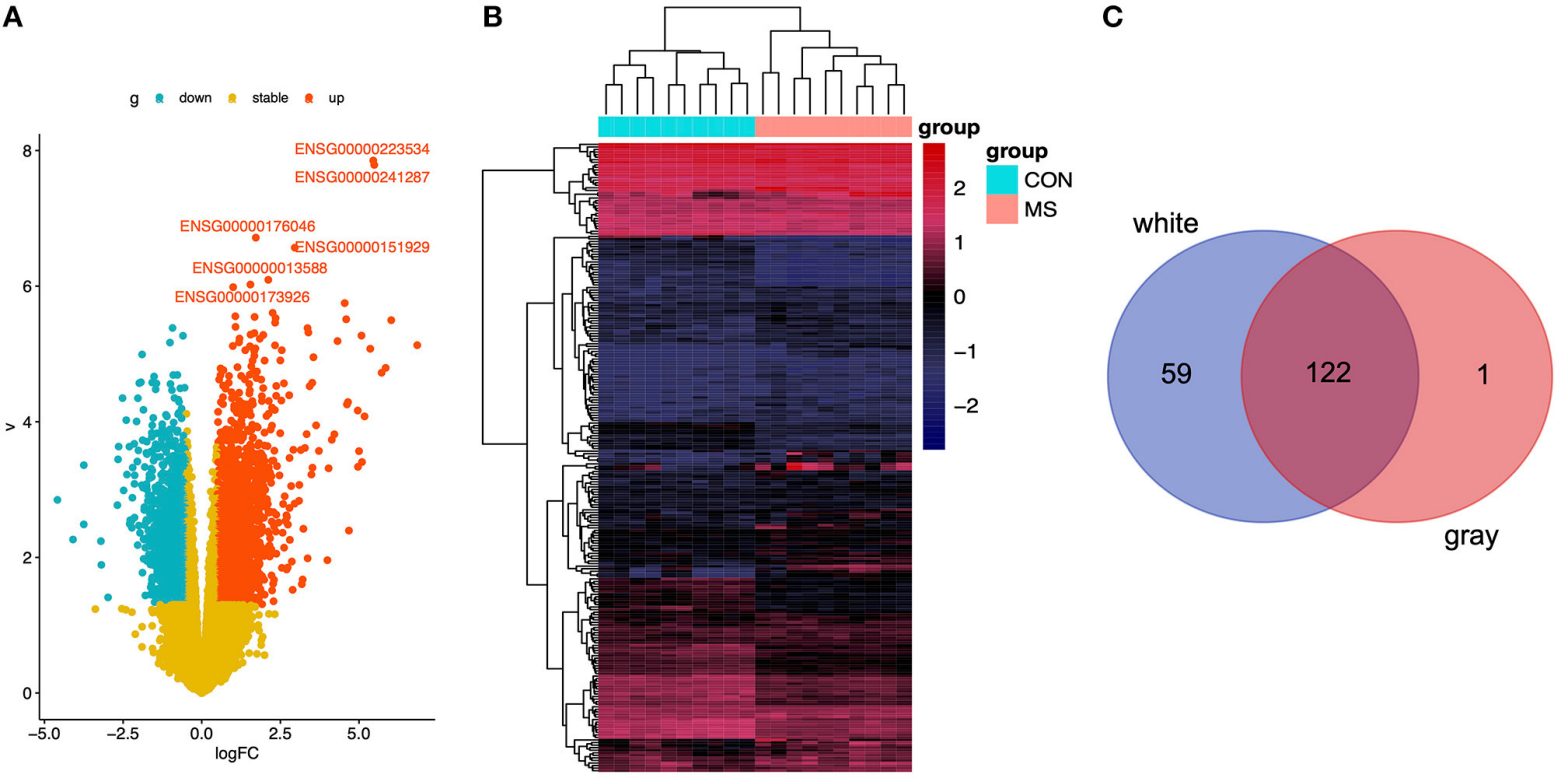
**(A)** Volcano plot of DEGs in WM. **(B)** Heatmap of DEGs in WM. **(C)** Using the Venn tools to find the overlap genes between downregulated genes in DEGs and genes in black module.

First, we calculated the DEGs between the WMLs and the WM normal control samples, the DEGs between the GMLs and the GM normal control samples. We found 181 DEGs in the WM group (*p* < 0.001, Figures 1A,B), 123 DEGs in the GM group (*p* < 0.001, Supplementary Figures 1B,C), and 122 overlapped DEGs were identified through overlapping analysis (Figure 1C).

### ssGSEA Analysis of MS Gene Expression Matrix in WM and GM

**Figure 2:**
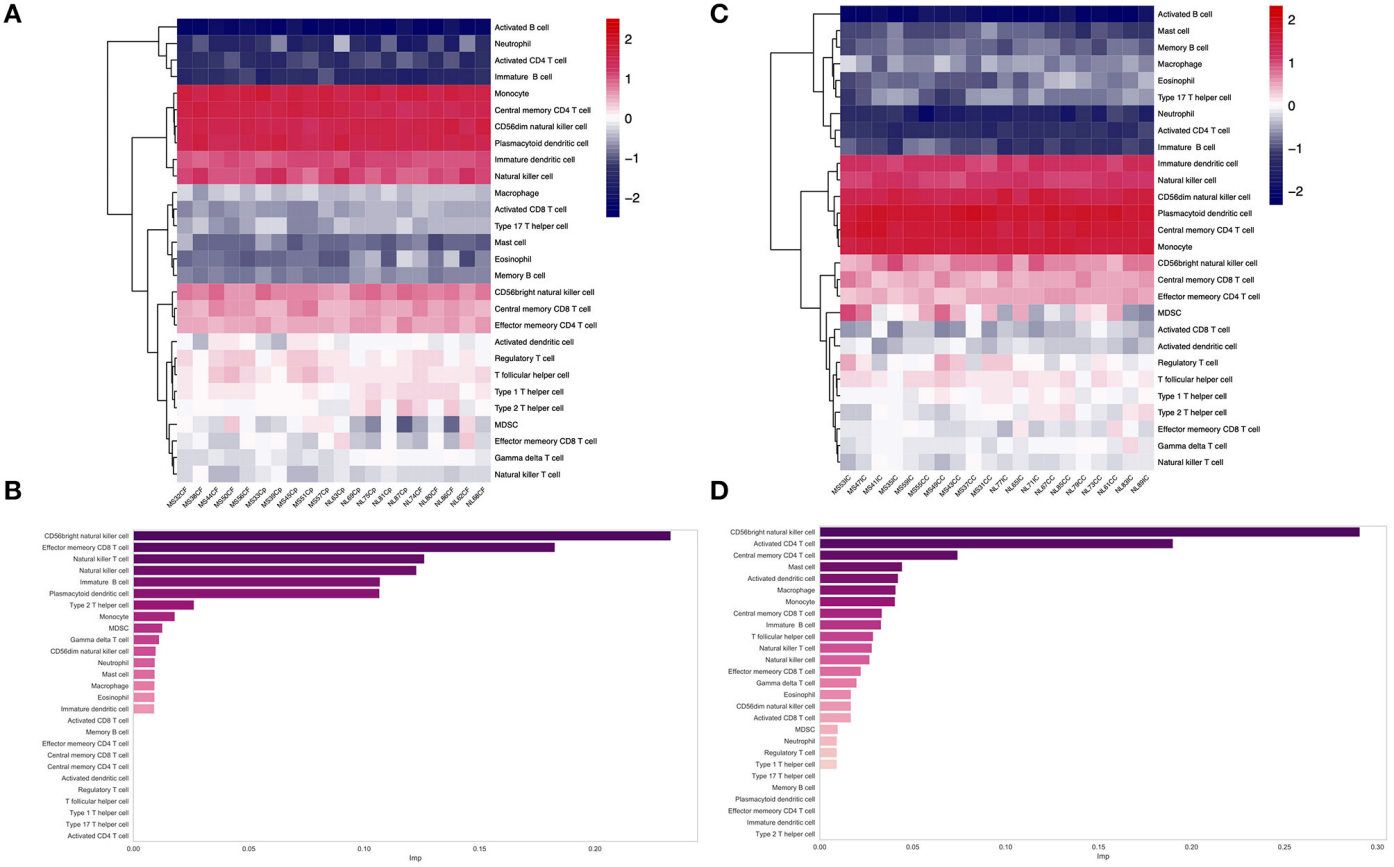
**(A,C)** Heatmap shows the ssGSEA scores of different gene sets in WM **(A)** and GM **(C)**. **(B,D)** The bar plot shows the relative importance of features (immune infiltration pathway) in the random forest classification model [**(B)** WM; **(D)** GM].

The ssGSEA analysis was performed on the gene expression matrix of 20 GM (10 GMLs vs. 10 normal) and 20 WM (10 WMLs vs. 10 normal). The analysis pathways mainly include 28 immune infiltration pathways, such as CD56 bright natural killer cell, effector memory CD8^+^ T cell, and memory B cell. As shown in Figures 2A,B, the scores in different samples are different. Furthermore, we found that there was a significant difference in the enrichment scores between the MS samples and the normal control samples, for example, the enrichment score for CD56 bright natural killer cell pathway in GM (Supplementary Figure 2, *p* < 0.001) and the enrichment score for effector memory CD8^+^ *T-*cell pathway in the WM (Supplementary Figure 3, *p* < 0.01) were significantly different. Finally, we compared the importance of the above relevant immune infiltration pathways in WM and GM, respectively (see the “Method). We found that in GM, CD56 bright natural killer cell pathway plays a key role, whereas, in WM, CD56 bright natural killer cell pathway is still important (Figures 2C,D). Furthermore, we also found that there is a certain negative correlation between the score of CD56 bright natural killer cell pathway and the scores of activated B-cell pathway, T follicular helper cell pathway, MDSC pathway, and so on in MS samples (Supplementary Figure 4).

### The Identification of Consensus Modules Across Different Brain Regions

The microarray data of 20 GM and 20 WM samples were read by R for hierarchical clustering. The consensus network of scale independence and mean connectivity analysis showed that when the weighted value was set to 14, the average degree of connectivity was close to 0, and the scale independence was about 0.8; therefore, the weighted value was set to 14 finally (Supplementary Figures 5A–C). WGCNA was performed to identify consensus modules. The comparison between GM set-specific modules and GM–WM consensus modules of the global co-expression network indicated that most WM modules were preserved in GM. All shared modules showed a significant overlap with the modules of the corresponding region-specific co-expression networks (Supplementary Figure 1D), demonstrating the similarity of clustering patterns in WM and GM.

### Relate the Consensus Modules With the ssGSEA Scores for Different Regions

**Figure 3:**
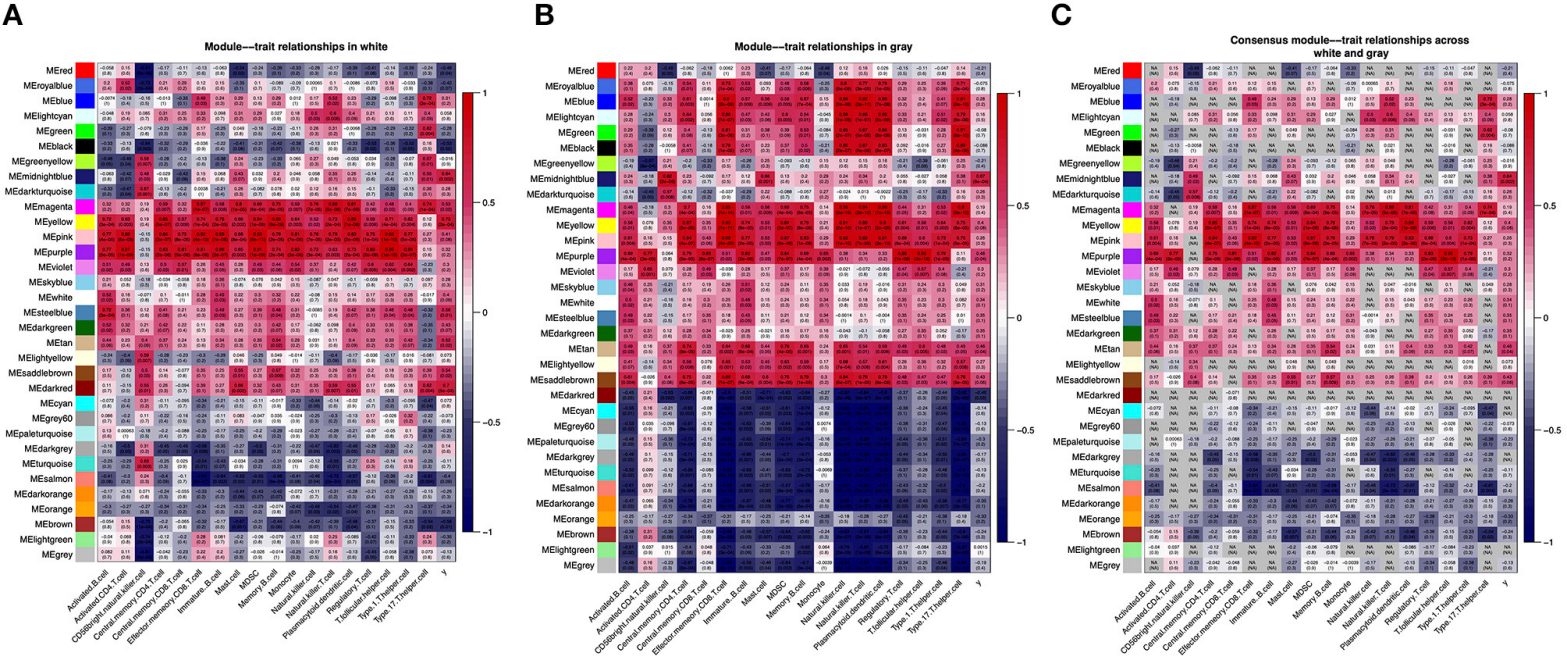
WM–GM consensus module construction. **(A)** Pearson correlation coefficients between the ssGSEA scores and module eigengenes (MEs) in WM dataset; numbers in brackets indicate the corresponding *p-*values. **(B)** Pearson correlation coefficients between the ssGSEA scores and module eigengenes in GM dataset; numbers in brackets indicate the corresponding *p-*values. **(C)** Pearson correlation coefficients between the ssGSEA scores and consensus module eigengenes; numbers in brackets indicate the corresponding *p-*values.

The module–feature relationship table showed the relationship between ssGSEA pathways (WM and scores of 18 pathways in Figure 3A, GM and scores of 18 pathways in Figure 3B) and consensus modules of different brain region datasets. The 2 relational heatmaps indicated a certain degree of similarity in the relationship of ssGSEA pathways and co-expression networks (Figure 3C). Furthermore, we found that the yellow, pink, purple, and magenta modules were correlated with most ssGSEA pathways significantly in each dataset, although the correlation coefficients and *p*-values between the two datasets were slightly different. In addition, we found that some modules in the two datasets had opposite correlation coefficients with the ssGSEA pathways, such as darkred and lightgreen modules. Finally, we took the lowest value of each correlation coefficient as the correlation coefficients of the comparison modules (Figure 3C). When the two values had opposite signs, we set the correlation coefficients of the comparison modules to NA. For instance, Figure 3A shows that the GM lightgreen module is negatively correlated with the effector memory CD8^+^ *T-*cell pathway, whereas in the WM dataset, the lightgreen module is positively correlated with the effector memory CD8^+^ *T-*cell pathway (Figure 3A), and the correlation between the consensus modules and the ssGSEA pathway is NA in the comparison table (Figure 3C). Next, we further analyzed the genes in midnightblue module because we found that there were 28 DEGs identified as hub genes in the midnightblue module (*GPR31, HOXD4, PAPPA, BPIFB4, LDHBP3, TMEM207*, etc.).

### Differential Consensus Module Eigengene Network Analysis Reveals Highly Preserved Network Structure

**Figure 4:**
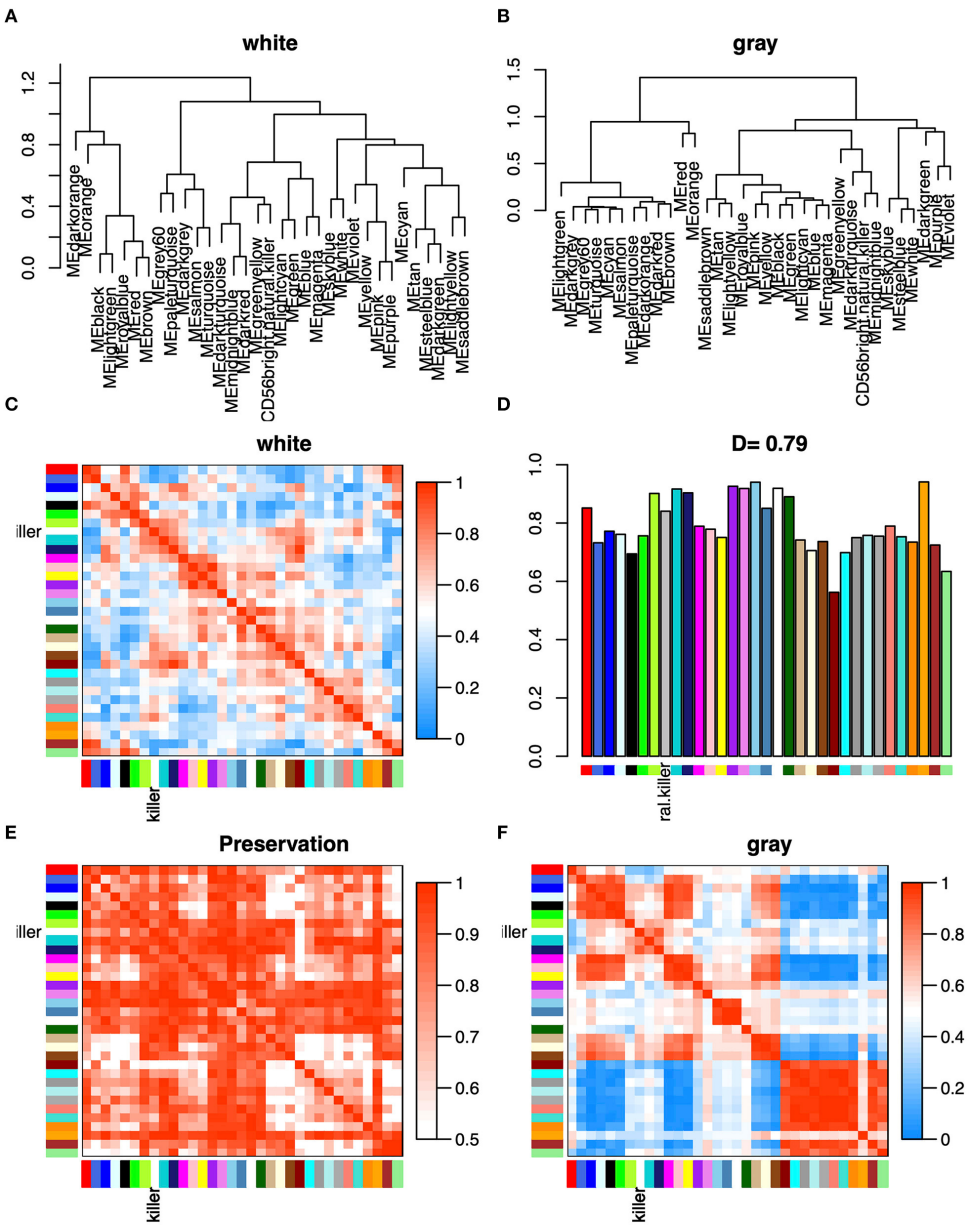
**(A,B)** Clustering dendrograms of consensus module eigengenes (MEs) for identifying meta-modules show the presence of similar major branching pattern in WM and GM eigengene networks. **(C,F)** The heatmap shows the eigengene adjacencies in WM and GM eigengene networks. Each row and column corresponds to an eigengene tagged by consensus module colors. Within each heatmap, red represents high adjacency (positive correlation) and blue represents low adjacency (negative correlation) as represented by the color legend. **(D)** Bar plot shows the preservation degree of each consensus eigengene as the height of the bar (*y*-axis), and each colored bar corresponds to the eigengene of the associated consensus module. The high-density value *D* (preserve WM and GM) = 0.79 indicates the high overall preservation between the WM and GM networks. **(E)** Adjacency heatmap of the preservation network between WM and GM consensus eigengene networks. The saturation of the red color indicates correlation preservation of WM and GM MEs.

In order to explore the overall preservation of correlation in the consensus module pairs between WM- and GM-specific networks, we analyzed the differential eigengene network (Figure 4). Based on the correlation of the pairwise consensus modules, we built eigengene network to evaluate the preservation of modules between WM and GM datasets. Figures 4A,B shows the overall preservation of the three networks is a positive correlation. The mean density of the two networks exceeded 0.79 in WM and GM datasets, demonstrating that the overall structures of the co-expression networks were similar between different brain regions. Furthermore, in Figure 4C, we found that the first meta-modules included red, brown, royalblue, lightgreen, and black module, and the second meta-modules included darkturquoise, midnightblue, darkred module, etc. However, these meta-modules also approximately existed in GM datasets. These results indicated that the differences between WM and GM may exist in the particular genes within the consensus networks.

### Identification of the Significant Modules and Genes in WMLs and GMLs and Construction of the Network

**Figure 5:**
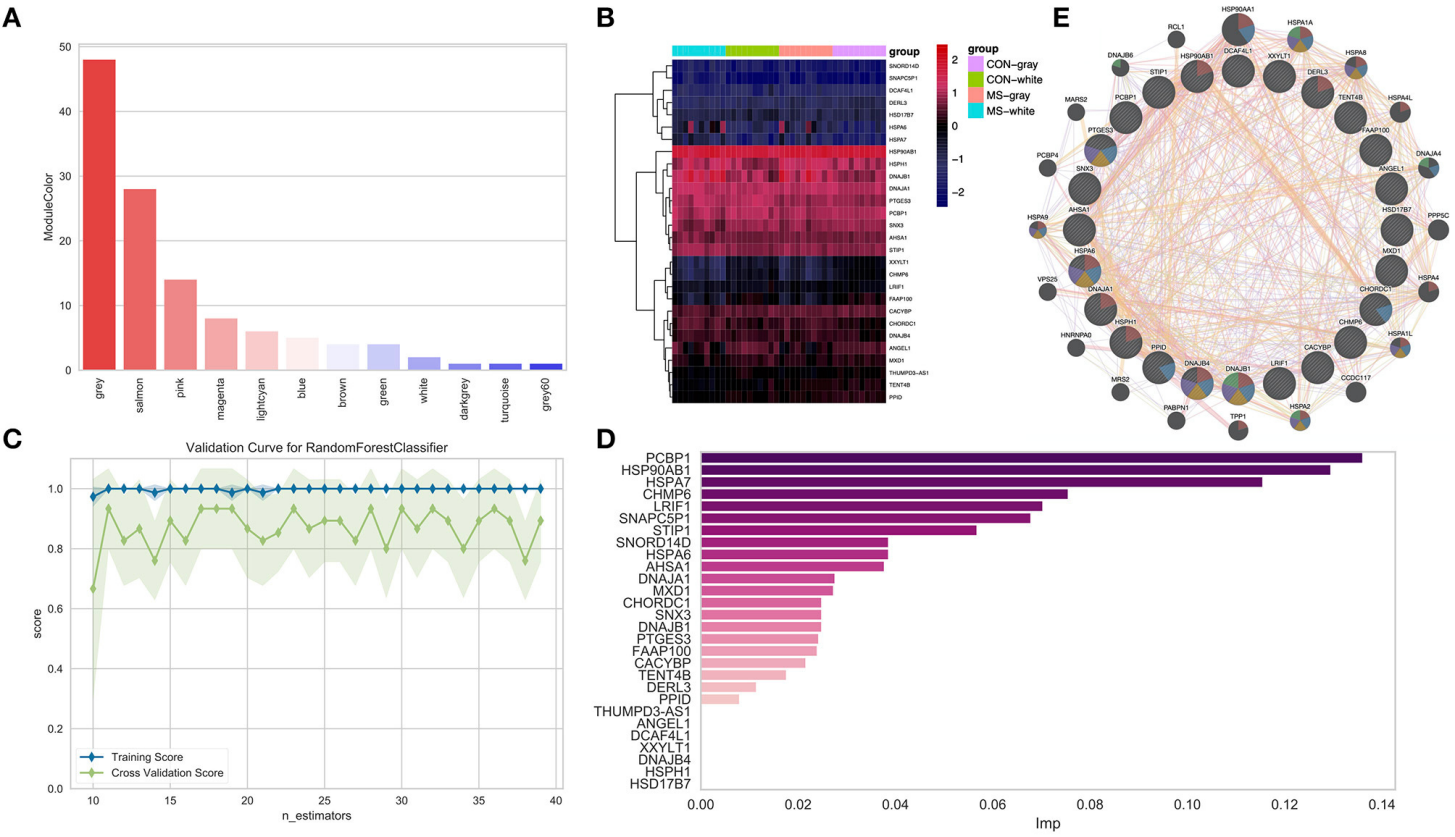
**(A)** Identifying the overlap genes between DEGs and genes in consensus modules. **(B)** Heatmap shows the expression of the overlapping genes in the midnightblue module. **(C)** The plot shows the changes of the f1 index with the changes of the max-feature in the training set and test set. **(D)** The bar plot shows the relative importance of features (genes) in the random forest classification model. **(E)** The PPI network of important genes *via* GeneMANIA.

In the above results, we obtained 122 overlapped DEGs, such as gene *GPR31 and so on*, and then, we identified the modules in which these genes were mainly distributed in. As shown in Figure 5A, we found that these genes were mainly distributed in the midnightblue module (28, DEGs), followed by the yellow module (9, DEGs) and purple module (8, DEGs).

To identify the most important genes in classifying MS and normal samples, the 28 genes in the midnightblue module (Figure 5B) were further filtered by the RF classification. Gene counts were inputted into the RF classifier model, and 40 (MS and normal) samples were shuffled. Furthermore, we used about 75% of the data as the training set and 25% of the data as the test set. Disease state was regarded as the classification target, and cross-validation was used to train the RF model. As shown in Figure 5C and Supplementary Figure 5D, in the test set, the ***f1*** = 0.8 and AUC = 0.86. Furthermore, we also obtained the relative importance of the genes in the trained RF model. As shown in Figure 5D and Supplementary Table 1, 21 important genes (*PCBP1, HSP90AB1, HSPA7, CHMP6, LRIF1, SNAPC5P1, STIP1, AHSA6*, etc.) were screened (imp > 0.01), and they were subjected to GeneMANIA to construct the protein–protein interaction (PPI) network (Figure 5E). We found that these genes only had a co-expression relationship, and few of them had an interaction between themselves.

## Discussion

Multiple sclerosis is an autoimmune neurological disease, and its pathological mechanism is still unclear (5, 6, 23). Furthermore, although the pathological changes in different brain regions are all mainly demyelinating lesions, the mechanisms are quite different. For instance, in the WMLs, the pathological mechanism is mainly the infiltration of immune cells, such as CD8^+^ T cells, whereas, in the GMLs, such immune cell infiltration is rare (3, 6, 7, 24). At present, although many studies focused on immune infiltration through ssGSEA, such as studying the role of immune infiltration in the tumor microenvironment and other autoimmune system diseases (25–27), it is rarely applied in studying MS in the brain. Therefore, integrating transcriptomics and ssGSEA to study the immune infiltration of MS plays a key role in elucidating its pathological mechanisms and searching for therapeutic targets. Therefore, we performed ssGSEA analysis on the pathways related to immune infiltration of WM and GM samples and found that 18 immune infiltration pathways were significantly different between the normal samples and the MS samples (Figure 2; Supplementary Figures 2, 3), such as the activated B-cell pathway and central memory CD4^+^ *T-*cell pathway. The memory B-cell pathway of immune infiltration pathways in the MS samples was significantly higher than those in the normal control samples, and these results are consistent with many previous studies (28–31). However, by the RF algorithm model, we identified that the most important pathway in the WM was the CD56 bright natural killer cell pathway, and in the GM, it remained the most important signal pathway (Figures 2C,D). Many researchers reported that, as the main type of natural killer cells, CD56 bright natural killer cell mainly played a role in inhibiting the *T-*cell signaling pathway in the disease progression of MS (32–34), thereby reducing its damage to the brain. In our study, we found that the score of CD56 bright natural killer cell signaling pathway in patients with MS is higher than that in the normal control samples, which may also be due to the resistance to *T-*cell damage. Furthermore, we also found that the scores of the CD56 bright natural killer cell pathway in the disease samples were negatively correlated with *T-*cell pathway scores (Supplementary Figure 4), which further indicated that the increase of CD56 bright natural killer cell pathway scores was due to the reduction of *T-*cell damage to the brain.

As a classic co-expression network algorithm, WGCNA is widely used in various diseases to find related hub genes. In this study, we perform the consensus WGCNA on the two brain region datasets, and then specific and opposite modules between WMLs and GMLs were identified. The mean density of the two networks exceeded 0.79 in consensus modules (Figure 4), demonstrating that the overall structures of the co-expression networks were similar in the WMLs and GMLs. We also found that there were 122 overlapped DEGs between WM and GM, and most of the DEGs in GM were kept in WM (Figure 1C) and modules with similar expression patterns between the two datasets, such as magenta, yellow, and pink modules. These results showed that the pathological mechanisms of the two were very analogous. In Figure 3, we found that the darkred module has opposite relationships in immune infiltration pathways, with the highest positive correlations with Mast cell in WM and negative correlations in GM, which indicated that there is an opposite relationship between the two gene expression patterns in the signaling pathway with Mast cell. These gaps may also be one of the reasons for the different pathological mechanisms of WM and GM.

Many studies are focusing on the mechanism of MS, and most studies showed that the pathological mechanisms in the GM and WM are similar to a certain degree (35–37). In this study, we found that there are certain differences and similarities between the pathological mechanisms of MS in GMLs and WMLs. Specifically, in our results, we found that the CD56 bright natural killer cell pathway plays the most important role in MS, regardless of whether it is in WM or GM. When the modules and the immune infiltration signaling pathways were analyzed for correlation, we found that the most relevant module to the CD56 bright natural killer cell pathway is the midnightblue module in GM (*r* = 0.82, *p* = 2e-06, Figure 3B). In order to better identify the hub genes in the midnightblue module, we overlapped the DEGs with the genes in midnightblue module and found 28 important hub genes, such as genes *GPR31, HOXD4, PAPPA, BPIFB4, LDHBP3, TMEM207*, etc. Similarly, we found the modules, which were most relevant to the CD56 bright natural killer cell pathway in WMLs, the darkturquoise module (*r* = 0.67, *p* = 0.001, Figure 3A), and we identified DEGs as hub genes, such as *PARVG, FCGR2A, HLA-DRB5*, and *OXTR* (Figure 3C). These results indicate that although the CD56 bright natural killer cell plays a key role in the WM and GM of MS, there are still some differences in the molecular mechanisms that mediate it.

In this study, we found that most of the DEGs belonged to the HSPs family (Figure 5B), which indicated that HSPs play a key role in MS. Although many studies reported that HSPs are involved in the pathological processes of many diseases in the brain, such as Alzheimer's disease, stroke, and brain trauma (38–40), in the pathological process of MS, the specific mechanism is still unclear. Some reports suggested that in the brain of MS, the level of HSPs is significantly increased (41, 42). However, whether these elevated HSPs are beneficial or harmful to patients with MS is still controversial. In our study, we also found that HSPs as important genes (Figure 5D) were mainly distributed in the midnightblue module, which further implied that HSP-related genes may play a synergistic effect with the genes in this module. In Figure 3C, we could discover that the immune infiltration pathway, which was most relevant to this module, was the CD56 bright natural killer cell pathway, and it implied that HSPs, which could inhibit the *T-*cell damage to the brain, may be involved in the CD56 bright natural killer cell pathway in MS.

## Conclusion

In this study, we compared the gene co-expression networks in GMLs and WMLs through WGCNA and ssGSEA methods and identified that the co-expression networks of the two had high similarities. Furthermore, we also identified the similarities and differences between the two co-expression networks in immune infiltration. The gene expression patterns of the two had similar expression in signal pathways, such as the activated B-cell pathway, central memory CD4^+^ *T-*cell pathway, and immature B-cell pathway. The differences between the two were mainly distributed in the CD56 bright natural killer cell pathway. Furthermore, we found that the HSPs, as the hub genes, were mainly distributed in the module, which was most relevant to the CD56 bright natural killer cell pathway, and it may be involved in antagonizing the damage caused by *T-*cells.

## Data Availability Statement

The datasets presented in this study can be found in online repositories. The names of the repository/repositories and accession number(s) can be found in the article/Supplementary Material.

## Author Contributions

KC: conceptualization, methodology, investigation, data curation, visualization, and writing the original draft. XZ: conceptualization and investigation. HT and GW: software. HG: resources and software. WY: data curation. SC: supervision. FW, DS, and JL: supervision, writing, reviewing, and editing. All authors contributed to the article and approved the submitted version.

## Conflict of Interest

The authors declare that the research was conducted in the absence of any commercial or financial relationships that could be construed as a potential conflict of interest.

## Publisher's Note

All claims expressed in this article are solely those of the authors and do not necessarily represent those of their affiliated organizations, or those of the publisher, the editors and the reviewers. Any product that may be evaluated in this article, or claim that may be made by its manufacturer, is not guaranteed or endorsed by the publisher.

## References

[1] Correale J Gaitán MI Ysrraelit MC Fiol MP. Progressive multiple sclerosis: from pathogenic mechanisms to treatment. Brain. (2017) 140:527–46. 10.1093/brain/aww258 27794524

[2] Dendrou CA Fugger L Friese MA. Immunopathology of multiple sclerosis. Nat Rev Immunol. (2015) 15:545–58. 10.1038/nri3871 26250739

[3] Kawachi I Nishizawa M. Significance of gray matter brain lesions in multiple sclerosis and neuromyelitis optica. Neuropathology. (2015) 35:481–6. 10.1111/neup.12216 26079808

[4] Ramagopalan SV Dobson R Meier UC Giovannoni G. Multiple sclerosis: risk factors, prodromes, and potential causal pathways. Lancet Neurol. (2010) 9:727–39. 10.1016/S1474-4422(10)70094-6 20610348

[5] Wildner P Stasiołek M Matysiak M. Differential diagnosis of multiple sclerosis and other inflammatory CNS diseases. Mult Scler Relat Disord. (2020) 37:101452. 10.1016/j.msard.2019.101452 31670010

[6] van der Poel M Ulas T Mizee MR Hsiao C-C Miedema SSM Adelia null. Transcriptional profiling of human microglia reveals grey-white matter heterogeneity and multiple sclerosis-associated changes. Nat Commun. (2019) 10:1139. 10.1038/s41467-019-08976-7 30867424 PMC6416318

[7] Prins M Schul E Geurts J van der Valk P Drukarch B van Dam A-M. Pathological differences between white and grey matter multiple sclerosis lesions. Ann N Y Acad Sci. (2015) 1351:99–13. 10.1111/nyas.12841 26200258

[8] Langfelder P Horvath S. WGCNA: an R package for weighted correlation network analysis. BMC Bioinformatics. (2008) 9:559. 10.1186/1471-2105-9-559 19114008 PMC2631488

[9] Liang J-W Fang Z-Y Huang Y Liuyang Z-Y Zhang X-L Wang J-L. application in Alzheimer's Disease. J Alzheimers Dis. (2018) 65:1353–1364. 10.3233/JAD-180400 30124448 PMC6218130

[10] Cui S Sun H Gu X Lv E Zhang Y Dong P. Gene expression profiling analysis of locus coeruleus in idiopathic Parkinson's disease by bioinformatics. Neurol Sci. (2015) 6:56. 10.1007/s10072-015-2304-0 25116258

[11] Chai K Liang J Zhang X Cao P Chen S Gu H. Application of machine learning and weighted gene co-expression network algorithm to explore the hub genes in the aging brain. Front Aging Neurosci. (2021) 13:707165. 10.3389/fnagi.2021.707165 34733151 PMC8558222

[12] Farhadian M Rafat SA Panahi B Mayack C. Weighted gene co-expression network analysis identifies modules and functionally enriched pathways in the lactation process. Sci Rep. (2021) 11:2367. 10.1038/s41598-021-81888-z 33504890 PMC7840764

[13] Panahi B Hejazi MA. Weighted gene co-expression network analysis of the salt-responsive transcriptomes reveals novel hub genes in green halophytic microalgae Dunaliella salina. Sci Rep. (2021) 11:1607. 10.1038/s41598-020-80945-3 33452393 PMC7810892

[14] Yuan H Lu J. Consensus module analysis of abdominal fat deposition across multiple broiler lines. BMC Genomics. (2021) 22:115. 10.1186/s12864-021-07423-6 33568065 PMC7876793

[15] Fang Q Wang Q Zhou Z Xie A. Consensus analysis via weighted gene co-expression network analysis (WGCNA) reveals genes participating in early phase of acute respiratory distress syndrome (ARDS) induced by sepsis. Bioengineered. (2021) 12:1161–72. 10.1080/21655979.2021.1909961 33818300 PMC8806251

[16] Barbie DA Tamayo P Boehm JS Kim SY Moody SE Dunn IF. Systematic RNA interference reveals that oncogenic KRAS-driven cancers require TBK1. Nature. (2009) 462:108–12. 10.1038/nature08460 19847166 PMC2783335

[17] Subramanian A Tamayo P Mootha VK Mukherjee S Ebert BL Gillette MA. Gene set enrichment analysis: a knowledge-based approach for interpreting genome-wide expression profiles. Proc Natl Acad Sci U S A. (2005) 102:15545–50. 10.1073/pnas.0506580102 16199517 PMC1239896

[18] Diboun I Wernisch L Orengo CA Koltzenburg M. Microarray analysis after RNA amplification can detect pronounced differences in gene expression using limma. BMC Genomics. (2006) 7:252. 10.1186/1471-2164-7-252 17029630 PMC1618401

[19] Ritchie ME Phipson B Wu D Hu Y Law CW Shi W. limma powers differential expression analyses for RNA-sequencing and microarray studies. Nucleic Acids Res. (2015) 43:e47. 10.1093/nar/gkv007 25605792 PMC4402510

[20] Warde-Farley D Donaldson SL Comes O Zuberi K Badrawi R Chao P. The GeneMANIA prediction server: biological network integration for gene prioritization and predicting gene function. Nucleic Acids Res. (2010) 38:W214–20. 10.1093/nar/gkq537 20576703 PMC2896186

[21] Franz M Rodriguez H Lopes C Zuberi K Montojo J Bader GD. GeneMANIA update 2018. Nucleic Acids Res. (2018) 46:W60–4. 10.1093/nar/gky311 29912392 PMC6030815

[22] Voskuhl RR Itoh N Tassoni A Matsukawa MA Ren E Tse V. Gene expression in oligodendrocytes during remyelination reveals cholesterol homeostasis as a therapeutic target in multiple sclerosis. Proc Natl Acad Sci U S A. (2019) 116:10130–10139. 10.1073/pnas.1821306116 31040210 PMC6525478

[23] Hemmer B Kerschensteiner M Korn T. Role of the innate and adaptive immune responses in the course of multiple sclerosis. Lancet Neurol. (2015) 14:406–19. 10.1016/S1474-4422(14)70305-9 25792099

[24] Waldman A Ghezzi A Bar-Or A Mikaeloff Y Tardieu M Banwell B. Multiple sclerosis in children: an update on clinical diagnosis, therapeutic strategies, and research. Lancet Neurol. (2014) 13:936–48. 10.1016/S1474-4422(14)70093-6 25142460 PMC4443918

[25] Ye L Zhang T Kang Z Guo G Sun Y Lin K. Tumor-infiltrating immune cells act as a marker for prognosis in colorectal cancer. Front Immunol. (2019) 10:2368. 10.3389/fimmu.2019.02368 31681276 PMC6811516

[26] Su J Long W Ma Q Xiao K Li Y Xiao Q. Identification of a tumor microenvironment-related eight-gene signature for predicting prognosis in lower-grade gliomas. Front Genet. (2019) 10:1143. 10.3389/fgene.2019.01143 31803233 PMC6872675

[27] Wang Z Yang H Zhang R Luo B Xu B Zhu Z. MEOX2 serves as a novel biomarker associated with macrophage infiltration in oesophageal squamous cell carcinoma and other digestive system carcinomas. Autoimmunity. (2021) 54:373–383. 10.1080/08916934.2021.1919880 34160343

[28] Danikowski KM Jayaraman S Prabhakar BS. Regulatory T cells in multiple sclerosis and myasthenia gravis. J Neuroinflammation. (2017) 14:117. 10.1186/s12974-017-0892-8 28599652 PMC5466736

[29] Lin C-C Edelson BT. New Insights into the Role of IL-1β in Experimental Autoimmune Encephalomyelitis and Multiple Sclerosis. J Immunol. (2017) 198:4553–4560. 10.4049/jimmunol.1700263 28583987 PMC5509030

[30] Maglione A Rolla S Mercanti SFD Cutrupi S Clerico M. The adaptive immune system in multiple sclerosis: an estrogen-mediated point of view. Cells. (2019) 8:E1280. 10.3390/cells8101280 31635066 PMC6829884

[31] Gross CC Schulte-Mecklenbeck A Rünzi A Kuhlmann T Posevitz-Fejfár A Schwab N. Impaired NK-mediated regulation of *T-*cell activity in multiple sclerosis is reconstituted by IL-2 receptor modulation. Proc Natl Acad Sci U S A. (2016) 113:E2973–2982. 10.1073/pnas.1524924113 27162345 PMC4889377

[32] Fehniger TA Cooper MA Nuovo GJ Cella M Facchetti F Colonna M. CD56bright natural killer cells are present in human lymph nodes and are activated by T cell-derived IL-2: a potential new link between adaptive and innate immunity. Blood. (2003) 101:3052–7. 10.1182/blood-2002-09-2876 12480696

[33] Laroni A Uccelli A. CD56bright natural killer cells: a possible biomarker of different treatments in multiple sclerosis. J Clin Med. (2020) 9:E1450. 10.3390/jcm9051450 32414131 PMC7291063

[34] Wang H Zeng Y Zhang M Ma H Xu B Jiang H. CD56brightCD16- natural killer cells are shifted toward an IFN-γ-promoting phenotype with reduced regulatory capacity in osteoarthritis. Hum Immunol. (2019) 80:871–877. 10.1016/j.humimm.2019.07.283 31326139

[35] Lee JK Kim N-J. Recent advances in the inhibition of p38 mapk as a potential strategy for the treatment of Alzheimer's Disease. (2017) 23:1287. 10.3390/molecules22081287 28767069 PMC6152076

[36] Frischer JM Bramow S Dal-Bianco A Lucchinetti CF Rauschka H Schmidbauer M. The relation between inflammation and neurodegeneration in multiple sclerosis brains. Brain. (2009) 132:1175–89. 10.1093/brain/awp070 19339255 PMC2677799

[37] Zhang J Giorgio A Vinciguerra C Stromillo ML Battaglini M Mortilla M. Gray matter atrophy cannot be fully explained by white matter damage in patients with MS. Mult Scler. (2021) 27:39–51. 10.1177/1352458519900972 31976807

[38] Thuringer D Garrido C. Molecular chaperones in the brain endothelial barrier: neurotoxicity or neuroprotection? FASEB J. (2019) 33:11629–39. 10.1096/fj.201900895R 31348679 PMC6902717

[39] Lackie RE Maciejewski A Ostapchenko VG Marques-Lopes J Choy W-Y Duennwald ML. The Hsp70/Hsp90 chaperone machinery in neurodegenerative diseases. Front Neurosci. (2017) 11:254. 10.3389/fnins.2017.00254 28559789 PMC5433227

[40] Salminen A Kaarniranta K Kauppinen A Ojala J Haapasalo A Soininen H. Impaired autophagy and APP processing in Alzheimer's disease: The potential role of Beclin 1 interactome. Prog Neurobiol. (2013) 106–107:33–54. 10.1016/j.pneurobio.2013.06.002 23827971

[41] Wali G Sue CM Mackay-Sim A. Patient-derived stem cell models in SPAST HSP: disease modelling and drug discovery. Brain Sci. (2018) 8:E142. 10.3390/brainsci8080142 30065201 PMC6120041

[42] Kim JY Kim JW Yenari MA. Heat shock protein signaling in brain ischemia and injury. Neurosci Lett. (2020) 715:134642. 10.1016/j.neulet.2019.134642 31759081 PMC6925329

