# Supplementary material for: The Application of Consensus Weighted Gene Co-expression Network Analysis to Comparative Transcriptome Meta-Datasets of Multiple Sclerosis in Gray and White Matter
Source: Front Neurol. 2022 Feb 24;13:807349. doi: 10.3389/fneur.2022.807349 (PMC8907380; doi:10.3389/fneur.2022.807349)
Supplement: Supplementary file 2 [file Data_Sheet_2.docx]

**Figure.S1 a)** Hierarchical clustering diagram of GM and WM samples. **b**) Volcano plot of DEGs in GM. **c)** Heatmap of DEGs in GM. **d)** Comparisons between GM set-specific modules and WM-GC consensus modules of the global co-expression network**.** The numbers in the table represented genes which were shared between GM modules and consensus modules. The color code of the table was -log(p), where p was the p value of Fisher's exact test of the two overlapped e modules. The darker the red, the more pronounced the overlap.

**Figure.S2** ssGSEA analysis of GM samples, comparisons of each signal pathway between the GMLs samples and the normal samples.

**Figure.S3** ssGSEA analysis of WM samples, comparisons of each signal pathway between the WMLs samples and the normal samples.

**Figure.S4 The relationship between the CD56 bright nature killer cell pathway scores and other immune infiltration pathways scores in MS sample. a)** Fitting relationship between activated B cell pathway scores and CD56 bright nature killer cell pathway scores. **b)** Fitting relationship between MDSC pathway scores and CD56 bright nature killer cell pathway scores. **c)** Fitting relationship between T follicular helper cell pathway scores and CD56 bright nature killer cell pathway scores.

**Figure.S5 a)** Scale independence of different of power. **b)** median connectivity of different soft power. **c)** mean connectivity of different soft power. **d)** ROCAUC plot of random forest.
